# Key Outcomes for a Vocational Rehabilitation Intervention for People With Multiple Sclerosis: A Nominal Group Technique Study

**DOI:** 10.1111/hex.70265

**Published:** 2025-04-10

**Authors:** Blanca De Dios Perez, Caolan Senior, Roshan das Nair, Denise Kendrick, Nikos Evangelou, Ian Newsome, Kate Radford

**Affiliations:** ^1^ Centre for Rehabilitation and Ageing Research, Queens Medical Centre University of Nottingham Nottingham UK; ^2^ NIHR Nottingham Biomedical Research Centre Nottingham UK; ^3^ Mental Health & Clinical Neurosciences School of Medicine, University of Nottingham Nottingham UK; ^4^ Institute of Mental Health, Nottinghamshire Healthcare Trust (Institute of Mental Health) Nottingham UK; ^5^ Health Division SINTEF Trondheim Norway; ^6^ Centre for Academic Primary Care, Lifespan and Population Health School of Medicine, University of Nottingham Nottingham UK; ^7^ Lay co‐author York UK

**Keywords:** consensus methods, multiple sclerosis, nominal group technique, outcome measurement, vocational rehabilitation

## Abstract

**Background:**

Job retention vocational rehabilitation (VR) interventions for people with multiple sclerosis (MS) can positively impact a person's independence and directly influence health and employment outcomes. However, evaluating the effectiveness of VR interventions can be challenging due to their highly individualised nature and the diverse range of potential health and employment outcomes that may be impacted.

**Aim:**

To identify the most important outcomes of a job retention VR intervention from the perspective of people with MS.

**Design:**

A nominal group technique (NGT) was conducted with people with MS using Microsoft Teams and Microsoft Forms. The study involved completing a demographic questionnaire, being introduced to VR, silently generating ideas, round‐robin discussions, ranking outcomes and reaching a consensus. NGT data were analysed using thematic analysis.

**Results:**

We conducted two NGTs involving 10 participants with MS. Nine outcomes were identified and ranked based on priority, relating to four themes: (1) Employer support and collaboration; (2) Empowerment; (3) Symptom management and progression and (4) Professional well‐being and performance.

**Conclusion:**

Improvements in employer support and symptom management were seen as key outcomes for VR interventions that could eventually lead to enhanced work performance and job retention for people with MS. Future research should explore how feasible it is to collect these outcomes to ascertain the impact of VR on employment over time. Due to the complexity of the outcomes identified, there may be a need to develop new outcome measures with improved validity and sensitivity to these interventions.

**Patient or Public Contribution:**

This study is part of a larger project co‐developed with three people MS. A lead patient and public involvement representative and lay co‐author reviewed the study topic guide to improve clarity and study focus and supported the group discussions and data analysis with input from the lead researcher.

**Abbreviations**
MS: Multiple sclerosis
NGT: Nominal group technique
PPI: Patient and Public Involvement
RCT: Randomised controlled trial
SD: Standard deviation
UK: United Kingdom
VR: Vocational rehabilitation

## 1 Introduction

Multiple sclerosis (MS) is a chronic neurological condition that affects young adults and can negatively impact their physical, cognitive and psychosocial well‐being [1, 2]. People with MS experience difficulties remaining in paid employment due to symptoms (e.g., fatigue, cognitive problems and pain) and environmental barriers, such as high workloads, attitudes toward disability and workplace inflexibility [3, 4, 5].

Vocational rehabilitation (VR) interventions aim to support people with illnesses or disabilities to remain, return or find new employment [6]. VR focused on job retention could positively impact the employment outcomes of people with MS by removing work‐related barriers through reasonable adjustments and supporting the person with symptom self‐management. Unfortunately, the evidence on the effectiveness of VR interventions for people with MS is inconclusive, partly due to the lack of effectiveness studies (including randomised controlled trials [RCTs]) and variability in outcome measures used [7]. RCTs are arguably the gold standard for measuring intervention effectiveness [8]; however, choosing the most relevant and meaningful outcomes is crucial for accurately assessing the true impact of these interventions.

Assessing the impact of VR interventions is complex. These interventions are highly individualised, require behaviour change from different stakeholders (e.g., the person with MS, the employer, colleagues and family members) and can affect multiple outcomes (e.g., job retention, return to work, self‐efficacy, etc.). Identifying and adopting standard outcome measures relevant to VR interventions could also facilitate understanding of the effectiveness of such interventions as they would enable comparison of evidence across trials [9].

Due to VR's highly individualised nature, understanding and prioritising outcomes most important to the individuals' receiving support is crucial. Patient‐centred outcomes can enhance participant engagement and active participation in the VR process. In addition, this approach aligns with person‐centred care to ensure the interventions address outcomes relevant to the population receiving the intervention beyond their clinical or medical needs [10].

Consensus techniques can help achieve this. One such approach is the nominal group technique (NGT) used to reach a consensus by sharing, discussing and voting on individually generated views on a given topic to achieve group consensus [11, 12]. This is a well‐established method that has previously been used to reach consensus on a wide range of healthcare topics, such as identifying important outcomes for pharmacy‐led primary care services [13], identifying preferences for end‐of‐life care for people with dementia [14] and identifying self‐care issues for people with diabetes [15], amongst others.

This study aimed to identify the most important outcomes for a job retention VR intervention from the perspective of people with MS for a future feasibility study.

## 2 Methods

This study was conducted between August and November 2024.

### 2.1 Study Design

The NGTs were organised remotely via Microsoft Teams and were video and audio recorded. We followed the guidance from Harvey and Holmes (2012). Three research team members were involved in the NGT (B.D.P., C.S. and I.N.). The lead researcher (B.D.P.) and a patient and public involvement (PPI) presentative (I.N.) jointly guided the NGT and used prompts and clarification. A research assistant (C.S.) guided the data collection and synthesis during the NGT.

First, the lead researcher explained the rationale and aims of the sessions to the participants. This was followed by an introduction to our previous research into VR for people with MS and the challenges of measuring the impact of these interventions. Subsequently, participants were presented with one research question: ‘What are the most important outcomes of a VR programme to help people with MS remain in paid employment?’

Participants were given 10 min to think on their own (*silent generation of ideas)* about outcomes that would be meaningful for them. Participants were asked to have their microphones on mute to avoid discussion and allow them the time to think and reflect on as many answers as possible. A Microsoft Form link was shared with the participants so they could write a list of statements reflecting relevant outcomes. The data were submitted anonymously, and only the research team could access the responses. An online form was chosen over other methods that allow participants to think about answers because people with MS experience memory and thinking difficulties, and remembering all their ideas can be challenging without writing them down.

The next stage, ‘*round robin’*, involved sharing the ideas (15 min). Participants were allowed to share the statements they submitted, one at a time, in rounds from the outcomes reported on their Microsoft Forms. All the statements referring to outcomes were compiled on a whiteboard for the participants to see. This was followed by a ‘clarification’ stage (30 min) to understand what participants meant by the outcomes reported. Some statements describing outcomes were grouped when they referred to the same idea. Participants could see on an online whiteboard how data were grouped based on similarities, which allowed them to keep track of ideas discussed and highlight disagreements.

Once the final list of outcomes was ready, participants were asked to rank the outcomes from most to least important using a prepopulated Microsoft Form link with the outcomes identified in the previous rounds. Participants had up to 10 min to rank the outcomes individually without group discussion.

### 2.2 Participants

We recruited people with MS in paid employment or who had recently left the workforce (in the last 12 months) following purposeful and convenience sampling, by sharing a study advertisement through social media posts and emails to a local group of people with MS who offer advice on MS studies conducted in the region and participants from previous research studies and contacting MS Together (an MS charity). Those interested in participating in the study contacted the lead researcher (B.D.P.) to be screened for eligibility and completed a written consent form and a brief questionnaire on clinical, personal and employment characteristics before participating in the NGT.

### 2.3 Materials

We used a PowerPoint presentation to facilitate the discussion and present the research questions during the NGT. Data were collected using Microsoft Forms, which participants accessed using a link and QR code according to their preferences.

### 2.4 Analysis

Data from the brief questionnaire were analysed using Excel and presented using descriptive statistics (mean, standard deviation [SD]).

### 2.5 NGT Data

Data collected from the NGT were imported into Excel. For the ranking exercise, all outcomes identified during the NGT were reviewed, and participants were asked to rank them from most to least important, with the first position being the most important outcome and the last being the least important one. The ranks were converted into scores, with higher ranks (i.e., the most important outcome) receiving a higher score and adding up the participants' scores for each outcome. The final ranking based on participants' preferences was presented to the participants for discussion.

Participants in the second NGT discussion received information regarding VR. As a pre‐elicitation technique, the nine outcomes from Group 1 were presented to participants in Group 2 [16]. Due to their similarity, participants in Group 2 decided to merge two outcomes from Group 1 and identified two further outcomes to be included in their ranking, leading to nine outcomes for the ranking exercise.

Data from the two NGTs were synthesised following Van Breda's (2005) guidance for analysing multiple‐group NGT data [16]. First, the data collected in the NGTs was transferred to an Excel spreadsheet. Then, the statements from each group were ordered based on NGT scores, with the most important outcomes from each group being at the top of the list. The mean score for each outcome was calculated by adding up the participant scores for the outcome, divided by the number of people. The top five outcomes, based on score, from each group were marked to progress to the following stages of analysis.

Step 3 involved content analysis of the data by categorising the statements collected during the NGT into themes based on meaning to ensure statements that relate to the same idea are grouped. A research team member confirmed the coding following the content analysis.

To identify the relative importance of the themes identified in the previous step, we counted how many ‘top five outcomes’ (i.e., identified in Step 2) were included per theme. Then, we counted how many ranked outcomes (i.e., nine per group) fell into each theme. Finally, we calculated the mean score for each theme by adding the mean score of each outcome and dividing by the number of statement statements per theme, as calculated in the earlier sub‐step. The values of the three sub‐steps were sorted in ascending order and given a rank to calculate the final rank by adding the three values [16]. This quantitative analysis allowed us to synthesise the quantitative data to identify the relative importance of each theme identified in Step 3. The final NGT findings are presented as a narrative description [16].

### 2.6 Quantitative Data

Agreement in ranking across participants for each group was tested using Kendall's *W*, and Friedman's test was used as a significance test if there were statistically significant differences in how outcomes were ranked by the two NGT groups. These analyses were conducted in SPSS v29.0 [17].

### 2.7 Qualitative Data

The audio files from the NGTs were transcribed verbatim and analysed using NVivo [18] following a thematic (Braun and Clarke [19]) analysis and a post‐positivist approach to identify the most important outcomes of VR interventions for people with MS. Thematic analysis involved data familiarisation, identifying initial codes, generating themes, review of the themes, and defining and naming the themes [19]. Two authors (B.D.P. and I.N.) were involved in the data analysis. The lead researcher (B.D.P.) conducted the data analysis and generated the themes used to group the outcomes identified in the two NGT groups. These findings were then presented to the lead PPI representative (I.N.), who reviewed the themes, descriptions and quotes selected based on his experience of attending the two NGT groups and provided feedback on how to improve the descriptions. The reviewed draft was then presented to the rest of the authors to refine the analysis and agree on the final themes.

## 3 Results

### 3.1 Participants

Ten people with MS [50% women; 50% men; age 46 (SD =11.72)] participated in the two NGTs (five participants in each NGT) (Table 1).

**Table 1:** Demographic, clinical and employment characteristics of participants.

MS participants characteristics (*n* = 10)	
Ethnicity*	***n* (%)**
Asian	1 (10%)
White British	8 (80%)
White European	1 (10%)
Relationship status	
In a relationship	9 (90%)
Single	1 (10%)
Type of MS	
Relapsing‐remitting MS	6 (60%)
Secondary progressive MS	3 (30%)
Unknown	1 (10%)
Years with MS [mean (SD)]	11.12 (9.62)
Education (years of education)	
College (10–12)	1 (10%)
A‐Levels (12)	1 (10%)
Degree (15)	5 (50%)
Post‐graduate (+16)	3 (30%)
Employment status	
Working	7 (70%)
Sick leave	1 (10%)
Unemployed	2 (20%)
SOC classification (*n* = 8)	
Level 2 (professional occupations)	2 (25%)
Level 3 (technical occupations)	4 (50%)
Level 4 (administrative and secretarial)	2 (25%)
Working hours (*n* = 8)	
Full‐time	5 (62.5%)
Part‐time	3 (37.5%)
Type of employer (*n* = 8)	
Private	3 (37.5%)
Public	5 (62.5%)
Organisation size (*n* = 8)	
Large (> 250 employees)	6 (75%)
Medium (50–249 employees)	2 (25%)
Industry (*n* = 8)	
Technology	3 (37.5%)
Business	2 (25%)
Security	1 (12.5%)
Government	1 (12.5%)
Education	1 (12.5%)

Abbreviation: SOC, Standard Occupational Classification.

* UK Ethnicity categories.

### 3.2 Outcomes

The participants identified 57 statements (35 Group 1; 22 Group 2) regarding outcomes recorded across the two NGTs in the first round of silent generation of ideas (Supporting Information File A). The 35 statements of Group 1 were synthesised into nine outcomes for the ranking exercise (Table 2).

**Table 2:** Outcome ranking by group.

Rank	Group 1	Group 2
1	Identifying and receiving reasonable adjustments	Identifying and receiving reasonable adjustments
2	Improve employer's understanding of MS	Improve employer's understanding of MS
3	Having a flexible work schedule	Working for as long as I wish
4	Improved understanding of legal rights	Improved confidence at work
5	Working for as long as I wish	Improved understanding of legal rights
6	Learning to pace workload throughout the day	Improved job satisfaction
7	Learning coping mechanisms for memory and thinking	Increase work productivity
8	Improved confidence at work	Learning to pace workload throughout the day
9	Learn more about MS	Learning coping mechanisms for memory and thinking

The outcome of ‘having a flexible work schedule’ was merged with ‘identifying and receiving reasonable adjustments’ because a flexible schedule is a type of reasonable adjustment. Participants also identified ‘increase work productivity’ and ‘improve job satisfaction’ as relevant outcomes, leading to nine outcomes for the ranking exercise. Table 2 presents the raw ranks per group (i.e., the outcomes ranked by group and the rank obtained, with top rank meaning most important).

We identified the top five outcomes for each group based on the group ranking, with higher scores representing a higher priority for the participants (Table 3).

**Table 3:** Ranking of top five outcomes across groups.

Group	Theme	Statement	Scores					Mean
1	Employer support and collaboration	identifying and receiving reasonable adjustments	9	9	8	8	5	7.8
1	Employer support and collaboration	Improve employer's understanding of MS	7	8	3	7	7	6.4
1	Employer support and collaboration	Having a flexible working schedule*	5	6	7	5	8	6.2
2	Employer support and collaboration	identifying and receiving reasonable adjustments	8	9	7	2	5	6.2
2	Employer support and collaboration	Improve employer's understanding of MS	4	4	9	9	1	5.4
1	Empowerment	Improved understanding of legal rights (*for the person with MS)*	8	4	5	9	3	5.8
1	Empowerment	Working for as long as I wish	6	5	9	4	4	5.6
2	Empowerment	Working for as long as I wish	7	6	3	8	9	6.6
2	Professional well‐being and performance	Improved job satisfaction	6	1	4	5	8	4.8
2	Managing MS symptoms and progression	Learning to pace workload throughout the day	9	8	5	4	3	5.8

*Note:* MS: Multiple sclerosis*Outcome excluded during NGT 2 due to overlap with other outcomes. Mean score: (sum ranking score % number of participants). Maximum mean score: 9.

The scores and rankings across both groups were synthesised to identify the top outcome for both groups (Table 4). The most important outcome for participants related to employer support and collaboration.

**Table 4:** Combined ranks across groups.

Theme	Number of outcomes per theme in top 5 outcomes from each group	Rank based on top 5 scores	Number of outcomes per theme from full outcome list	Rank based on total outcome scores	Mean score of statements per theme*	Rank based on mean scores	Final rank
Employer support and collaboration	5	4	5	2.5	6.4	4.00	10.5
Empowerment	3	3	6	4	5	3	10
Managing MS symptoms and progression	1	1.5	5	2.5	3.72	1	5
Professional well‐being and performance	1	1.5	2	1	4.7	2	4.5

*Note:* Final rank: Relevance score based on combined rank scores (rank based on top 5 scores + rank based on total outcome scores + rank based on mean scores).

*The mean score of each statement is presented in Table 3.

### 3.3 Kendall's *W* and Friedman's Test

The Kendall's *W* demonstrated a small to moderate agreement between participants in both groups (Group 1 *W* = 0.459, *p* = 0.019; Group 2 *W* = 0.396, *p* = 0.037), suggesting that there was individual variation in the preferences for the outcomes ranked. There was a statistically significant difference in the Friedman tests in how outcomes were ranked (Group 1: *χ* ^2^ = 18.34, *p* = 0.019; Group 2: *χ* ^2^ = 17.81, *p* = 0.037).

### 3.4 Thematic Analysis

Four themes were identified from the group discussion. The names of the nine outcomes identified in the study are in italics.

#### 3.4.1 Theme 1: Employer Support and Collaboration

The most important theme for the participants emphasised the crucial role of employers in supporting job retention and making the person with MS feel valued at work. Participants believed that their employer had to collaborate with them to find working agreements that allowed them to have an improved work experience.

Improving *employer's understanding of MS* was considered one of the most important outcomes. They repeatedly reported that employers need to be educated about the variety of MS symptoms, their variability, progression and impact on work ability.

Researcher: What would you like employers to learn about MS? […] Participant 6: So what is MS, What does it mean? What is a disability? What does it mean? And what does progression mean today? I can walk today, but maybe tomorrow I can't. So, what does progression mean?

This was particularly important to our participants with MS because of the lack of understanding around invisible symptoms and how MS fluctuates, which led to challenges at work following organisational policies (e.g., sick leave) and employers' preferences (e.g., not allowing breaks during the day).

‘I think if you challenge that [employers' understanding of MS] first, the other outcomes will just follow. Like I think it's very hard to touch on some of the things that I want to achieve at work, without employers actually at least trying to understand what MS is anyway.’

Participant 10

Participants experienced challenges *identifying and receiving reasonable adjustments* at work. They believed that reasonable adjustments could help them with their performance difficulties. However, they encountered challenges obtaining support because employers are the stakeholders who decide what is ‘reasonable’.

‘The problem with that is how subjective reasonable adjustments are. Because who defines what's reasonable? You might be asking for something that you think is completely reasonable, like working from home 2 days a week. But they'll turn around and say to you, no, that's not happening.’

Participant 3

One recurrent reasonable adjustment highlighted by the participants was having a flexible working schedule (i.e., adaptable hours and changes in the start and end of working hours) to manage the fluctuating nature of MS.

#### 3.4.2 Theme 2: Empowerment

Our participants aimed to become empowered at work, and three outcomes were included within this theme.

Participants expressed the importance of *working for as long as one wishes* as their top priority. This was seen as having the option to control their employment choices and avoid being forced out of the workforce when they believed they could still work.

‘I mean, yeah, working as long as you can. For the last 2 years, I have been asked, “Have you thought about medical retirement?” And my answer is no, I will work as long as I can. Because I don't want to give up work. I want to get up. I want to have a purpose.’

Participant 1

When participants discussed the topic of remaining at work, they also considered the idea of receiving support to move to an alternative role that was more suited to their skills. Therefore, for some, this would involve looking for alternative jobs, developing new skills or completing further education.

‘It should always be an option [further education] rather than just giving up work…because as we know when living with MS…when things are negative, then you lose that motivation, and then I end up getting more symptomatic.’

Participant 5

An *improved understanding of legal rights* and relevant legislation (e.g., the Equality Act 2010) was seen as essential to empowering them to have conversations with their employers and advocate for their needs at work.

‘Let's say there is a law that requires some accommodation of your needs, so you know…your confidence could be affected by a number of different things, but that would be good in terms of outcomes for maybe stress and so on because you're going to be less worried about your positioning if you know what your rights are.’

Participant 3

Participants also expressed an interest in having *improved confidence at work*. Gaining this confidence would allow them to handle difficult conversations about work and negotiate with their employers about their needs at work.

‘You know, a bit of confidence in knowing maybe that your employer will be prepared to accept you are doing things in a different way or at a different time.’

Participant 4

#### 3.4.3 Theme 3: Managing MS Symptoms and Progression

Participants reflected on challenges that they experience at work and the need to have strategies to manage or mitigate the impact of MS symptoms at work.

Higher fatigue levels can lead to reduced productivity and engagement in work commitments. Although the participants were using strategies to manage their fatigue, higher workloads and a lack of workplace flexibility increased their challenges at work.

‘My fatigue predicts productivity. I mean, I do 8 hours a day, but I'm a lot more productive at the start of the day.’

Participant 1

Another common challenge related to cognitive difficulties (e.g., concentration, word‐finding difficulties and memory problems). These affected the confidence and productivity of the participants with MS at work and therefore suggested as an outcome ‘*learning coping mechanisms for memory and thinking’*.

‘I worked all over before I had to stop, mainly for cognitive issues. But when I was there, people would have quite different understandings of the work that I could do or how.’

Participant 8

#### 3.4.4 Theme 4: Professional Well‐Being and Performance

Overall, participants were interested in having *improved job satisfaction* and *increased work productivity*.

A series of factors affected the job satisfaction of the participants with MS at work. Sometimes, they felt disengaged from their role because they thought they were not valued or treated fairly compared to how other coworkers were treated.

‘For my workload to be reduced. I have to be absent, but I'm not sick. I just I'm just slower.’

Participant 6

Participants were keen on remaining productive and wanted to thrive at work, provided that their employers made some accommodations to ensure they achieved their best performance.

‘Symptoms that come and go over time. So, like you say, 1 day you're OK; next, you're not that productive…that has got to be so recognised…You know, it's not like every day you're going to be expected to achieve the same level of performance.’

Participant 8

## 4 Discussion

This study identified nine important outcomes of a job retention intervention to support people with MS at work. These outcomes will inform the final selection of outcomes for a feasibility study. The NGT findings underscore the multifaceted nature of VR for people with MS. The findings illustrate the need to address individual (e.g., symptoms and progression) and environmental (e.g., employers' attitudes and knowledge) barriers to job retention.

The top outcome selected by the participants referred to ‘employer support and collaboration’. This was prioritised because participants believed having a more flexible work environment would allow them to be more productive and reduce the impact of their MS at work. This aligns with previous evidence that suggests that work inflexibility is one of the main factors contributing to job loss for people with illnesses or disabilities [20].

There was variability in the ranking of the outcomes between groups, suggesting that participants had diverse preferences for what they would consider valuable outcomes at work, which impacted their outcome prioritisation exercise. Further exploration of how employment characteristics inform the prioritisation of outcomes could be explored in future research.

Most discussions focused on the impact of employers, their views and their lack of understanding of MS at work. This was seen as one of the key priorities; in fact, participants shared 24 statements (out of 56) about this theme. These results align with the literature exploring what services or support people with MS want as part of their VR services, highlighting that receiving support to manage their performance and the expectations of their employers is essential to help them at work [21].

Employers are the gatekeepers to providing reasonable adjustments, and a better relationship between employer and employee is directly related to improved provision of reasonable adjustments [22, 23]. Feeling supported at work is directly linked to reducing stressful working conditions, which can lead to improved productivity outcomes [22].

Unfortunately, previous research into VR for people with MS has demonstrated significant challenges in engaging employers in these interventions and research overall [24, 25]. This is also seen in VR for other health conditions, such as stroke survivors trying to return to work [26]. The challenges with employer engagement are related to people with MS not wanting to include them in the interventions because of fear of discrimination and/or having a complicated relationship with their employers [24, 25]. There may be other reasons why employers do not engage more in these programmes, but these are less well understood. This suggests the need to develop interventions targeted at supporting employers to support employees with illnesses and disabilities at work to ensure they have the skills needed to discuss work and health if an employee has a disability.

Other key outcomes identified referred to symptom management, particularly cognition and fatigue. There is evidence that self‐reported work ability in people with MS is influenced by fatigue, disability levels and occupation, with those with higher disability and fatigue levels reporting the worst self‐reported work ability [27]. This suggests that interventions that offer symptom management and the provision of reasonable adjustments could improve work ability levels in people with MS.

The complexity of the outcomes identified in this NGT study reflects the multifaceted nature of VR for people with MS. Some outcomes identified (e.g., employer understanding, confidence of the person with MS, symptom management, etc.) are interconnected and require change at an individual and environmental level. Therefore, there may be challenges associated with measuring these outcomes and understanding how they change over time.

Even though the outcomes were ranked based on priority for people with MS, it is essential to understand that VR follows an iterative problem‐solving process to change attitudes and modify the work environment to limit the factors that result in work disability [28, 29]. Removing work disability requires time to change attitudes and the environment. Therefore, some outcomes will require a longer timeline to achieve a meaningful change (i.e., remaining at work for as long as you wish) than others (i.e., improved understanding of legal rights).

Interestingly, there were no outcomes relating to improving physical or mental health. Only one participant raised this outcome during the discussion, but it did not receive sufficient attention and relevance to progress to the next stage. This is because it was seen more as a ‘healthcare’ topic, as opposed to the purpose of a job retention intervention. Even though work is good for health [30], and for people with MS, working full‐time is associated with improved cognitive and physical ability and reduced fatigue levels [31]. This may suggest that people with MS might not always perceive a clear relationship between their work and health outcomes. Previous research indicates that people with MS do not always bring their employment issues to healthcare professionals, or these are only discussed in a moment of crisis [32, 33]. Future research should explore outcomes of interest from the perspective of people with MS, their employers and healthcare professionals to have a more comprehensive understanding of outcomes that are relevant for all key stakeholders engaged in supporting people with illness or disability in employment.

### 4.1 Strengths and Limitations

The main strength of this study is the integration of mixed‐methods research to address the research question. By synthesising the quantitative and qualitative data regarding the outcomes identified by the participants, we gained a better understanding of relevant outcomes for VR interventions. Another strength is the inclusion of a sample of participants with MS with diverse employment characteristics to capture the needs and preferences of people from different professional backgrounds.

However, this study is not without limitations. This study's main limitation is the sample size (*n* = 10). Even though we recruited participants with diverse employment (employed, on sick leave and recently unemployed), from a range of jobs with different levels of seniority and from secretarial or administrative to professional occupations, this sample is arguably small. The smaller sample size was due to recruitment issues. Even though 10 participants were recruited for the first NGT, only five attended the session. This led to the research team organising a second event with seven participants recruited and only five attending the second NGT. Participants provided reasons for not attending the NGT groups, such as feeling unwell, unexpected work commitments and technological difficulties. Regardless of the recruitment challenges, we felt that the number of participants recruited was sufficient for the purpose of this study and aligns with previous NGT studies that have included between 8 and 13 participants [34, 35, 36].

Another limitation of the study refers to the use of convenience sampling; while this approach facilitates the recruitment of participants with diverse experiences, it can also lead to selection bias, because those with more positive or negative employment experiences may be more willing to participate.

Regardless of the recruitment challenges, this study presents valuable insight into VR outcomes relevant to people with MS, for example, by highlighting the relevance of the employer's role in supporting people with illness and disabilities at work.

## 5 Conclusion

A job retention programme focused on employer collaboration, empowerment, effective symptom management strategies and integration of robust support systems could enable people with MS to thrive in the workplace and avoid premature job loss.

Future trials in MS and VR should consider measuring the outcomes identified in this study to ensure they target meaningful outcomes and improvements for people with MS.

## Author Contributions

**Blanca De Dios Perez:** conceptualisation, investigation, funding acquisition, writing – original draft, methodology, validation, visualisation, writing – review and editing, software, formal analysis, project administration, data curation, resources. **Caolan Senior:** methodology, writing – review and editing, data curation. **Roshan das Nair:** conceptualisation, funding acquisition, methodology, validation, writing – review and editing, supervision, investigation. **Denise Kendrick:** conceptualisation, funding acquisition, investigation, methodology, validation, writing – review and editing, supervision. **Nikos Evangelou:** conceptualisation, investigation, funding acquisition, methodology, validation, writing – review and editing, supervision. **Ian Newsome:** supervision, conceptualisation, investigation, funding acquisition, methodology, writing – review and editing. **Kate Radford:** supervision, conceptualisation, investigation, funding acquisition, writing – original draft, methodology, writing – review and editing, project administration.

## Ethics Statement

This study received favourable ethical approval from the University of Nottingham (FMHS 223‐0624). Before the group discussion, eligible participants were briefed on the study's objectives and completed a written consent form before data collection.

## Conflicts of Interest

B.D.P. has received funding from the Neurology Academy (speakers bureau) to deliver lectures on vocational rehabilitation for people with MS. R.d.N. has received funding (speakers bureau) from Novartis, Biogen and Merck for delivering lectures on psychological aspects of MS and cognitive screening and rehabilitation in MS.

## Supporting information

Supplementary File A.

## Data Availability

The data from the nominal group technique questionnaires that support this study's findings are available in this article's supporting material. The group discussion transcripts are available upon reasonable request to the corresponding author. The data are not publicly available due to privacy or ethical restrictions.
