# Supplementary material for: Key Outcomes for a Vocational Rehabilitation Intervention for People With Multiple Sclerosis: A Nominal Group Technique Study
Source: Health Expect. 2025 Apr 10;28(2):e70265. doi: 10.1111/hex.70265 (PMC11985894; doi:10.1111/hex.70265)
Supplement: Supplementary file 1 — Supplementary File A. [file HEX-28-e70265-s001.docx]

**Supplementary File A – Outcomes shared by participants of NGT**

Table S1 presents the statements reported by the participants of the two NGTs grouped according to overarching theme and outcome category. These statements were used to develop the broader outcome groups that participants subsequently ranked in the study.

**Table S 1 Examples of outcomes shared by NGT participants.**

| **Overarching Theme** | **Outcome** | **Statements** |
| --- | --- | --- |
| **Employer’s support and collaboration** | **Identifying and receiving reasonable adjustments** | Ability to have disability leave for treatment medical appointments |
|  |  | Ability to have leave or flexible hours within a role. |
|  |  | Improved flexibility in role |
|  |  | Improved flexibility in role (e.g. work activities or flexible work times) |
|  |  | Having on-site support to help with queries / enquiries on an issue / idea. |
|  |  | ease of access [Improved workplace accessibility] |
|  |  | Flexibility in working schedule |
|  |  | Greater flexibility for appointments, etc. |
|  |  | To receive appropriate reasonable adjustments |
|  |  | Flexibility for appointments |
|  |  | Colleagues’ acceptance of impact of reasonable adjustments |
|  |  | More comprehensive understanding of potential adjustments that are possible and that might be suitable |
|  | **Improved employer’s understanding of MS** | Most important is a mutual understanding between employers and employee on sustainability within the role |
|  |  | Having access to a support network (colleagues / supervisor / manager / etc |
|  |  | Going forward, Work design fully recognises the needs and limitations of pwMS |
|  |  | Collaborating with colleagues - i.e. more heads are better than one. |
|  |  | Employers to understand MS and the types of MS |
|  |  | Education to the workforce of what MS actually is, and how people can be supported |
|  |  | Employers to understand needs of those with MS and how they can adjust meetings to accommodate |
|  |  | Trust |
|  |  | Proactive suggestions (e.g. rebalance work week) from employer |
|  |  | Ability for candid discussions |
|  |  | knowledge about MS progression and impact on performance |
|  |  | Employers: Understanding of your condition. MS is different for each person |
|  |  | Understanding of MS by colleagues: Fluctuation, differences between people. |
| **Empowerment** | **Improved confidence at work** | Confidence |
|  | **Improved understanding of legal rights** | Access to legal/employment rights and support because when you’re ill, you’re using all your energy to recover and don’t have much left to fight to stay employed |
|  |  | Help to locate and understand HR policies within an organisation. |
|  |  | What are my rights/benefits etc |
|  |  | Support from OT or legal professionals where required |
|  |  | Availability of resources and support on employment rights |
|  |  | Legal Rights & Employer obligations |
|  | **Working for as long as I wish** | Control your destiny |
|  |  | Opportunities to adopt alternative role |
|  |  | Empowerment |
|  |  | Working for as long as you can. |
|  |  | To work effectively for as long as possible (length of time working) |
| **Managing MS symptoms and progression** | **Learning to pace workload throughout the day** | making the day comfortable to avoid fatigue |
|  |  | Having access to fresh air |
|  |  | Having short breaks to stetch legs and not experience stiffness / cramp by sitting in the same place for too long |
|  |  | Understanding of fatigue and impact of it |
|  | **Learning coping mechanisms for memory and thinking** | Focus on a specific goal without being confused by another target |
|  |  | Cannot concentrate if there is too much noise |
|  |  | Having time to think about scenarios / situations in which I am in. |
|  |  | Taking time to read and understand an issue |
|  |  | Having quiet to work on individual tasks |
|  |  | Length of time able to work effectively (How much time we can work effectively on a day) |
| **Professional well-being and performance** | **Improved work productivity** | Accept fluctuating performance throughout the week. |
|  |  | To reduce the amount of sickness absence due to MS (number of sick days) |
|  |  | Caseload not reduced if you are present at work - Reduced caseload for those working slower |
|  |  | To be more effective in role (achieve more positive performance appraisals) |
|  | **Improved job satisfaction** | To improve job satisfaction |
|  |  | Better work appraisal outcomes |
|  |  | Career Progression |
|  |  | Successful career development |
|  |  | Positive performance feedback from line managers |
